# Protein-Based Systems for Topical Antibacterial Therapy

**DOI:** 10.3389/fmedt.2021.685686

**Published:** 2021-06-24

**Authors:** Raj Kumar Thapa, Krister Gjestvang Grønlien, Hanne Hjorth Tønnesen

**Affiliations:** Section for Pharmaceutics and Social Pharmacy, Department of Pharmacy, University of Oslo, Oslo, Norway

**Keywords:** antibacterial therapy, chronic infected wounds, proteins, topical formulations, wound healing

## Abstract

Recently, proteins are gaining attention as potential materials for antibacterial therapy. Proteins possess beneficial properties such as biocompatibility, biodegradability, low immunogenic response, ability to control drug release, and can act as protein-mimics in wound healing. Different plant- and animal-derived proteins can be developed into formulations (films, hydrogels, scaffolds, mats) for topical antibacterial therapy. The application areas for topical antibacterial therapy can be wide including bacterial infections in the skin (e.g., acne, wounds), eyelids, mouth, lips, etc. One of the major challenges of the healthcare system is chronic wound infections. Conventional treatment strategies for topical antibacterial therapy of infected wounds are inadequate, and the development of newer and optimized formulations is warranted. Therefore, this review focuses on recent advances in protein-based systems for topical antibacterial therapy in infected wounds. The opportunities and challenges of such protein-based systems along with their future prospects are discussed.

## Introduction

The integrity and function of the skin as a physical permeation barrier are crucial for protecting against the external environment, including particles, exogenous chemicals, and microorganisms (1). The skin is considered the largest human organ and is composed of two main layers; epidermis and dermis (2). The epidermis is further divided into five separate layers (in order from most superficial to deepest: stratum corneum, stratum lucidum, stratum granulosum, stratum spinosum, and stratum basale) (3). The barrier function and transportation of compounds into the skin can be attributed to the most superficial layer, stratum corneum, and is governed by a brick-like structure of corneocytes (4). The second skin layer is the dermis, which is responsible for the strength and elasticity of the skin. The dermis consists mainly of fibroblast cells and fibrous proteins, such as collagen and elastin (5). The skin has a microbiota composed of numerous bacteria and fungi with an essential role in the protection against invading pathogens (6). A breach of the skin barrier can disturb the balance between commensals and pathogens, resulting in skin disease and infection (7).

The barrier function of the skin can be reduced by trauma, such as accidental injury, cuts, scrapes and burns, or skin disease. Disruption of the skin can lead to impaired protection against the external environment, including the risk of infections and compromised immunity (8). Diseased skin, such as common acne and atopic dermatitis is associated with dysbiosis between common commensal species, resulting in inflammation and epidermal barrier impairment (6). Changes in the skin microbiota can also be the result of diseases, such as immunodeficiency diseases and diabetes (9, 10).

**Figure 1:**
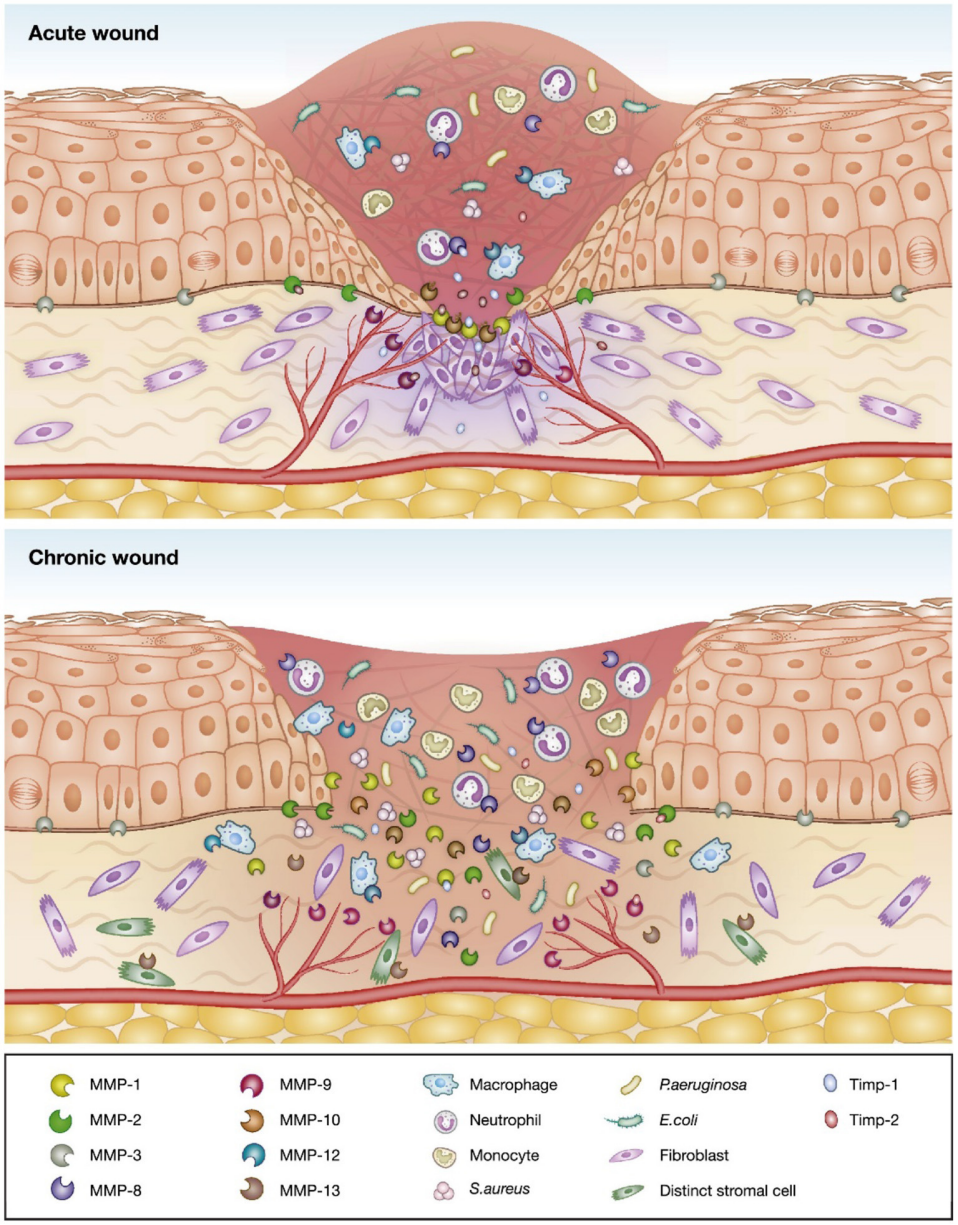
Schematic of the differences between acute and chronic wounds illustrated by an increased imbalance between proteolytic enzymes and their inhibitors, and invasion of opportunistic bacteria leading to delayed wound healing in chronic wounds. Adapted with permission from Krishnaswamy et al. (12).

Wounds that do not heal by an orderly sequence of events and within a predictable amount of time are defined as chronic (11). The pathophysiology of chronic wounds is characterized by a disturbance in the normal physiological conditions, including the colonization of opportunistic pathogens (Figure 1). Bacterial infections may cause wound deterioration, which will slow down the healing process and prevent wound closure (13, 14). Opportunistic pathogens, such as the Gram-positive bacterium *S. aureus* and the Gram-negative bacterium *P. aeruginosa* are prevalent in chronic wounds (13, 14) which can form biofilms characterized by an aggregation of immobilized bacterial cells in an adhesive extracellular matrix (15, 16). Biofilm formation affects the wound healing process by delaying epithelialization and granulation tissue formation. Such wounds express lower levels of inflammatory markers, thus disturbing the mechanisms of recovery (17). Further, the production of destructive enzymes and toxins may promote a chronic inflammatory state of the wound and prevent the healing process (18).

Topical drug delivery can be used for delivery of drugs through the skin, i.e., transdermal drug delivery, or formulations may be applied for local action, i.e., retention of the active ingredient on the skin surface or within the epidermal layers without systemic absorption (19). Topical antibiotics and antiseptics are commonly prescribed to treat superficial bacterial infections (20, 21). However, clinical guidelines have been focused on systemically or intravenously administered antibiotics (20). The clinical usage of topical antibacterial treatment has been limited to superficial skin infections, such as impetigo, chronic wounds and burns, and the prevention of postsurgical wound infections and minor traumatic wound infections (20). Conventional antibacterial agents used for topical treatment include antiseptics, such as chlorhexidine, triclosan, and hydrogen peroxide, and antibiotics such as mupirocin, bacitracin, polymixin B, neomycin, gentamicin, silver sulfadiazine, and fusidic acid (20, 22). A major concern regarding the use of antibiotics includes the potential resistance development by the infecting bacteria (23). Thus, an optimal formulation is required to effectively kill the pathogens and lower the risk of resistance development. Commercial formulations for topical antibacterial therapy have been based on conventional formulation techniques, such as emulsions (creams and lotions), ointments, and hydrogels, and moisture-retentive dressings, such as hydrocolloids and poly-urethane foams (20, 22, 24). The major advantages of topical formulations are their local effects without systemic absorption leading to fewer side effects, ease of application, moisturizing properties, low cost of production, and inexpensiveness for patients (25). However, the topical application may interfere with the wound healing process and the normal microbiota. Further, the residence time of the active ingredient may be too short for a proper effect, and the dose accuracy may be low. Some patients may find the application of the formulations painful (22, 25). Thus, there is a need for modified formulations suitable for topical applications.

## Proteins: Roles in Wound Healing and Topical Antimicrobial Therapy

Wound healing is a complex biological process involving damaged tissue replacement with a living one (26). The tissue integrity is restored as a result of interactions of platelets, cells (e.g., monocytes/macrophages, neutrophils, fibroblasts, keratinocytes, and endothelial cells), and extracellular matrix (ECM) components (27, 28). The ECM not only provides essential physical scaffolding for the cellular constituents but also initiates crucial biochemical and biomechanical cues that are required for tissue morphogenesis, differentiation, and homeostasis (29). The ECM is composed of two main classes of macromolecules: proteoglycans and fibrous proteins (30). Collagen, elastin, fibronectin, and laminin are the main fibrous proteins in the ECM (31). Collagen is the most abundant fibrous protein within the interstitial ECM (constitutes up to 30% of total protein mass) with important functions including regulation of cell adhesion, provide tensile strength, support chemotaxis and migration, and directs tissue development for wound healing (32). Collagen further associates with elastin fibers that provide recoil to tissues undergoing repeated stretch (33). Fibronectin protein is involved in directing the organization of interstitial ECM and has a crucial role in mediating cell attachment and function (34).

Some proteins and peptides have inherent antimicrobial properties. Such antimicrobial proteins and peptides have been studied for their potential in the treatment of bacterial wound infections in addition to their wound healing properties (35). Antimicrobial peptides (AMPs) are the host-defense peptides produced by animals, plants, fungi, bacteria, and protozoa. Detailed information on potent AMPs for topical application in wounds is available elsewhere (36). Several AMPs (e.g., Pexiganan, Omiganan, Lytixar, Dalbavancin, and Brilacidin) with potential as antibacterial agents are in different phases of clinical trials for future commercial developments (37). Additionally, there are some enzymes (e.g., lysozyme, phospholipase A_2_) that possess antibacterial properties. These proteinaceous molecules have a different mechanism of actions e.g., lysozyme breaks the bond between the N-acetylglucosamine (NAG) and N-acetylmuramic acid (NAM) which make up the peptidoglycan backbone (38), and phospholipase A_2_ penetrate the bacterial cell wall and hydrolyzes the phospholipids in the bacterial cytoplasmic membrane (39).

Besides the inherent antimicrobial properties of proteins and peptides, they can be used in the preparation of formulations for the treatment of topical infections in wounds. Different plant- and animal-derived proteins have been studied for their potential in wound healing. Protein wound dressings, such as hydrogels, sponges, and sheets have advantages in wound healing by adhering to the tissue and absorbing excess wound exudate. Protein formulations may further protect the wound against secondary infections and retain a moist wound healing environment (40–42). Such protein formulations are prepared by different methods including desolvation, nanoprecipitation, coacervation, emulsification, self-assembly, layer-by-layer assembly, and electrospinning (43). Among these methods, electrospinning has gained recent interest in the preparation of protein-based systems for topical drug delivery. The electrospinning process generates ultrathin fibers by utilizing electrostatic force to the protein or polymeric solution (44), and these fibers can be used for the modification and control of drug release (45).

## Proteins and Their Formulations for Topical Antibacterial Therapy of Infected Wounds

**Figure 2:**
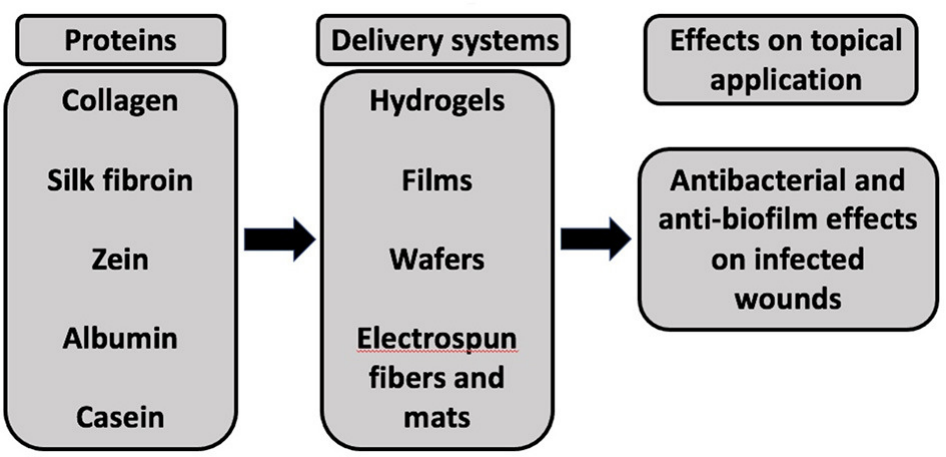
A schematic representation of different proteins used as delivery systems for antimicrobial agents to exhibit antibacterial and anti-biofilm effects upon application to infected wounds.

Proteins such as collagen, silk fibroin, zein, albumin, and casein can be used as a carrier (e.g., hydrogels, films, wafers, and electrospun fibers, and mats) for antimicrobial agents to support antimicrobial action and promote healing of infected wounds (Figure 2).

The protein-based systems loaded with antimicrobial agents and investigated for their antibacterial activity are summarized below:

### Collagen

Collagen is the main component of connective tissue composed of a triple helix formed by three α-chains (repeating triplets of the amino acids glycine-X-Y where X and Y are often proline and the imino acid hydroxyproline, respectively) (46). It has been isolated from bovine, porcine, equine, avian, and aquatic species (47–49). Collagen derived from aquatic sources (fish and jellyfish) and plant-derived recombinant collagen has further been investigated as potential alternatives to mammalian collagen to mitigate issues on transmissible diseases and religious preferences (49–51). Additionally, collagen from marine sources has also gained attention because of the AMPs derived from their collagen. An example of such AMP is collagencin which is derived from fish collagen (52). The exogenous collagen is biocompatible, biodegradable, non-toxic, and weakly antigenic compared to other natural polymers (53). Collagen can cross-link and self-aggregate to form fibers with high tensile strength and stability (46). These fibers can be formulated into different scaffolds for wound dressing. Collagen has an ability to attract proteases, which have been exploited in commercial products acting as sacrificial substrates, protecting the endogenous collagen in the wound bed (54, 55). Gelatin is a hydrolyzed form of collagen which can also be utilized for topical application in infected wounds (56). Collagen is a highly versatile material with potential applications for burn/wound cover dressings (57). Recently, the drug delivery properties of collagen have been widely studied. Collagen-based drug delivery systems for potential topical application in infected wounds include microspheres based on gelatin, collagen scaffolds, collagen hydrogels, collagen/collagen-synthetic polymer hydrogels, and collagen films. Most of the commercial formulations of collagen are available only as skin substitutes for wound healing application but do not possess antibacterial activity (58). There are only two commercial collagen-based antimicrobial dressings containing silver as an antimicrobial agent: collagen/oxidized regenerated cellulose (ORC)-silver (Promogran® Prisma, Systagenix) and collagen-silver (Puracol® Plus Ag+, Medline) (59). However, collagen dressings containing other antimicrobial agents are still lacking in the commercial market. Different studies exploring the potential of collagen-based systems as topical antibacterial agents have been published. The examples of such collagen-based systems for topical antibacterial therapy of infected wounds are presented in Table 1. The combination of wound healing and controlled drug release properties of collagen can effectively kill the infecting bacteria and assist in the wound healing process. However, a potential disadvantage is the protease-mediated growth of *S. aureus* under nutrient-limited conditions while using collagen in an antibacterial system (68). Therefore, rigorous *in vitro* and *in vivo* studies are required to optimize and establish the potential use of collagen-based systems as topical antibacterial therapy for infected wounds.

**Table 1:** Collagen-based systems for topical antibacterial therapy of infected wounds.

**Collagen-based formulation**	**Active ingredient**	**Preparation method**	**Additional polymer/ nanoparticle**	**Treatment purpose**	**Target microorganism**	**Outcomes**	**References**
Film	Pexiganan	Simple mixing of active ingredient and collagen, and air drying	-	Infected rat wound model (*in vivo*)	*S. aureus* and *P. aeruginosa*	The sustained pexiganan release was observed for 72 h; *in vivo* wound bacteria inhibition was 3-5 fold higher compared to open wound or blank collagen film	(60)
Scaffold	Silver sulfadiazine	Simple mixing of silver sulfadiazine loaded alginate microspheres in pepsin-solubilized collagen	Alginate microspheres	*In vitro* antibacterial studies	*K. Pneumoniae, E. coli, P. aeruginosa*, and *S. aureus*	The collagen scaffold controlled drug release for up to 72 h. Minimum inhibitory concentrations (MIC) and minimum bactericidal concentration (MBC) were *K. Pneumoniae* (MIC: 32 μg/mL and MBC: 40.2 μg/mL), *E. coli* (MIC: 32 μg/mL and MBC: 40.2 μg/mL), *P. aeruginosa* (MIC: 44.8 μg/mL and MBC: 51.2 μg/mL), *S. aureus* (MIC: 57.6 μg/mL and MBC: 57.6 μg/mL)	(61)
Scaffold	Doxycycline	Simple mixing of doxycycline loaded gelatin microspheres in pepsin-solubilized collagen	Gelatin microspheres	Infected rat wound model (*in vivo*)	*P. aeruginosa*	Early subsidence of infection (99.9%) by day 9 for collagen scaffold treated infected wounds whereas, for the control group, the microbial load exceeded 10^3^ CFU even on day 15	(62)
Hydrogel	Lysostaphin	Mixing of solubilized chitosan and collagen, and subsequently incorporating lysostaphin into the purified hydrogels	Chitosan	Infected rabbit burn wound model (*in vivo*)	Methicillin resistant *S. aureus* (MRSA)	The MIC of chitosan-collagen hydrogel incorporating lysostaphin (CCHL) was 0.053 U/mL. No bacteria were detected in the wounds by the second week of CCHL application on MRSA infected third-degree burn wounds.	(63)
Wafers	Gentamicin	Electrospinning of polycaprolactone/collagen nanofibers and subsequent coating with micelles composed of polypeptide-based block copolymer	Polycaprolac-tone/polypeptide based block copolymer micelle	*In vitro* antibacterial studies	*S. aureus*	The gentamicin-loaded wafers were able to kill >99.99% of *S. aureus*	(64)
Nano-composite	Gentamicin sulfate and sodium rifamycin	Resuspending drug loaded silica particles in collagen gel	Silica particles	Infected rat wound model (*in vivo*)	*S. aureus*	Sustained antibacterial effects over 10 days *in vitro* and 2 log reduction in the bacterial population following treatment with nanocomposites	(65)
Scaffold	Silymarin and silver nitrate	Mixing of chitosan and collagen along with silymarin, and addition of bi-layer scaffolds of silver incorporated chitosan and collagen	Chitosan	No *in vitro* or *in vivo* antibacterial studies were performed	-	Antibacterial studies are warranted for further assessment of *in vitro* and *in vivo* antibacterial activity of the scaffold	(66)
Scaffold	Zinc oxide nanoparticles	Homogenized mixing of zinc oxide nanoparticles in collagen gel	Zinc oxide nanoparticles	*In vitro* antibacterial studies	*S. aureus* and *E. coli*	Growth inhibition zones of the scaffold were comparable to those obtained for tested antibiotics (methicillin, trospectomycin, and ceftolozane/tazobactam for *S. aureus*; and colistin, streptomycin, and rifampin for *E. coli*.	(67)

### Silk Fibroin

Silk fibroin is a biologically-derived protein polymer purified from domesticated silkworm (*Bombyx mori*) or non-mulberry (*Antheraea assama*) cocoons (69) with unique properties including biocompatibility (70), biodegradability (71), mechanical strength (72), high water and oxygen uptake (73), and excellent properties for drug delivery and tissue engineering (74). The regulation of beta sheet content (crystallinity) can control the degradation time course of silk implants from days to years (75, 76). Furthermore, the incorporation of sensitive compounds (e.g., proteins) without loss of bioactivity is possible in silk attributable to its ability to be processed in aqueous systems under mild and ambient conditions of temperature and pressure (77, 78). There are reports on the use of silk fibroin as an antibacterial biomedical nanotextile for wound dressing (79) and as a controlled release system for antibiotics (80). However, there are no commercial antibacterial products based on silk fibroin. Several studies exploring the topical antibacterial potential of silk fibroin-based systems have been published. The examples of such systems for topical antibacterial therapy of infected wounds are presented in Table 2. Rigorous studies are warranted to establish the *in vivo* antibacterial potential of topical silk fibroin-based systems for commercial development in the treatment of infected wounds.

**Table 2:** Silk fibroin-based systems for topical antibacterial therapy of infected wounds.

**Silk fibroin-based formulation**	**Active ingredient**	**Preparation method**	**Additional polymer/ nanoparticle**	**Treatment purpose**	**Target microorganism**	**Outcomes**	**References**
Hydrogel	Ampicillin	Prepared from an aqueous solution of silk fibroin protein polymer and bulk loaded ampicillin	-	Infected mouse wound model (*in vivo*)	*S. aureus*	Sustained ampicillin release was observed for 72 h; *in vivo* wound bacteria inhibition was 20 fold higher compared to untreated wound	(80)
Electrospun mats	Polyethylene-imine (PEI)	PEI and silk fibroin were dissolved in formic acid to achieve a final concentration of 15% w/v	PEI	*In vitro* antibacterial studies	*S. aureus* and *P. aeruginosa*	Potent antibacterial activity against both bacteria and their complete inhibition	(79)
Film	Ciprofloxacin Amoxicillin Nystatin	Simple mixing of antibiotics to 8% w/v silk fibroin solution prior to casting	-	*In vitro* antibacterial studies	*S. aureus, P. aeruginosa, K. pneumonia, A. baumannii, E. coli*, and *C. tropicalis*	The antibacterial activity of free drug solutions was maintained by the mucoadhesive silk fibroin films	(81)
Composite film	Gold nanoparticles	Simple mixing of silk fibroin solution and gold nanoparticles prior to casting	Gold nanoparticle	Infected rat wound model (*in vivo*)	Multidrug resistant *E. coli*	Complete inhibition of multidrug resistant bacteria *in vitro* and efficiently combat infection in *in vivo* wound model	(82)
Layer-by-layer deposited nanofibers	Lysozyme	Composite nanofibrous mats were prepared from mixed solutions of silk fibroin and nylon 6 using electrospinning. Lysozyme and collagen were alternately assembled on the prepared mats by layer-by-layer deposition.	Lysozyme and collagen alternate deposition of 10 layers on silk fibroin and nylon 6 composite nanofibrous mat	*In vitro* antibacterial studies	*E. coli*, and *S. aureus*	>80% and >98% reduction in the viable count of *E. coli*, and *S. aureus*	(83)

### Zein

Zein is a plant protein found in maize endosperm (84) composed of nonpolar and uncharged amino acids glutamine, leucine, proline, and alanine (85). The solubility of zein is determined by its amino acid composition and are accordingly classified as α, β, γ, and δ zein (86). Zein is a biocompatible and biodegradable polymer with potential applications in biomedical and pharmaceutical fields (87). It is widely used in the food and pharmaceutical industry attributable to its ability to form a tough, glossy coating with antibacterial activity (84, 88). Zein can be prepared as nanoparticles, microspheres, films, fibers, and composites with other natural polymers (87) using the evaporation-induced self-assemblage mechanism for controlled delivery of drugs (89). So far, there are no commercial products of zein for topical antibacterial therapy of infected wounds. Published literature suggest the potential of zein-based systems as potent topical antibacterial formulations. Examples of such systems are presented in Table 3.

**Table 3:** Zein-based systems for topical antibacterial therapy of infected wounds.

**Zein-based formulation**	**Active ingredient**	**Preparation method**	**Additional polymer/ nanoparticle**	**Treatment purpose**	**Target microorganism**	**Outcomes**	**References**
Electrospun composite mat	Streptomycin sulfate	Electrospinning of blended mixtures of polyurethane, cellulose acetate, and zein	Polyurethane and cellulose acetate	*In vitro* antibacterial studies	*S. aureus, B. subtilis, E. coli, S. typhimurium*, and *V. vulnificus*	Antimicrobial activity was observed against both Gram-positive and Gram-negative bacteria	(90)
Electrospun nano-composite mat	Silver nanoparticles	Electrospinning of zein solution and concurrent synthesis of silver nanoparticles *in situ* into the mats	-	*In vitro* antibacterial studies	*S. aureus* and *E. coli*	Antimicrobial activity was observed against both bacterial strains	(91)
Fibrous matrices	Tetracycline	Electrospinning of blended mixtures of zein, poly-ε-caprolactone, and tetracycline	Poly-ε-caprolactone	*In vitro* antibacterial studies and *ex vivo* pig skin infection model	Methicillin resistant *S. aureus* (MRSA)	A 75% reduction in MRSA biofilm was achieved *in vitro* and an 81% reduction in bacterial colony forming units (cfu) was achieved in the *ex vivo* pig skin infection model	(92)
Electrospun nanocomposite scaffold	Gum arabic	Electrospinning of blended mixtures of zein, poly-ε-caprolactone, and gum arabic	Poly-ε-caprolactone and gum arabic	*In vitro* antibacterial studies	*S. aureus* and *E. coli*	Antibacterial activity was observed only against *E. coli*	(93)
Multilayer wound dressing membrane	Gentamicin	Preparation of zein film using a mixture of zein and gentamicin and addition of an electrospun layer over it	-	*In vitro* antibacterial studies	*S. aureus* and *P. aeruginosa*	Sustained gentamicin release and potent antibacterial activity in both the bacteria	(94)

### Albumin

Albumin is the most abundant plasma protein (35–50 g/L human serum) with a molecular weight of 66.5 kDa (95). It is produced by the liver and has an important role in maintaining the osmotic pressure of plasma and transporting endogenous compounds (proteins, cholesterol, and bile pigments) (96). Albumins (bovine serum albumin and human serum albumin) are widely utilized for a variety of clinical and biomedical research applications attributable to their advantageous intrinsic properties such as biocompatibility, biodegradability, low immunogenicity, and non-toxicity (95, 97). Albumin possesses non-specific protein adsorption property that has led to its design as an antimicrobial material to reduce the adhesion of pathogenic bacteria (98, 99). Coating of a polystyrene membrane with human serum albumin inhibited biofilm formation by *E. coli* (100) and pneumococcal strains (101). Nevertheless, the inhibitory effect of albumin is still limited to certain bacterial species (102) and can even stimulate bacterial growth in other species (100, 101). Such inhibitory effect of albumin can be utilized in the preparation of topical films or patches with albumin coatings to promote anti-biofilm activity once applied to susceptible wounds. The triggered release behavior of albumin nanoparticles (103) can be potentially utilized for the on-demand antibacterial release upon application to infected wounds. Furthermore, albumin coatings on nanoparticles can facilitate the targeting and trafficking during drug delivery (104). Similar strategies can be used for topical application of albumin coated nanoparticles to facilitate uptake inside the cells infected with bacteria for the induction of antibacterial effects. There are limited literature about the use of albumin-based systems for topical antibacterial therapy. Further research in this field is warranted to explore the potential use of albumin-based systems for the topical antibacterial therapy.

### Casein

Casein is a milk phosphoprotein that constitutes a major (≈ 80%) proportion of cow milk protein content (105). It is a proline-rich, amphiphilic protein, which can self-assemble into micellar structures (106). Casein is inexpensive, non-toxic, and heat-stable, and can form film attributable to its random coil conformation (107). Although there are reports on different formulations of casein [nanoparticles (108), microparticles (109), micelles (110), films (111), hydrogels (112)] in drug and enzymatic delivery; there are negligible studies about casein-based antimicrobial preparations for topical applications. The high availability, biocompatibility, and biodegradability properties of casein make it a suitable protein for the potential delivery of bioactive molecules and antimicrobial agents for topical delivery in tissue engineering and wound healing. The film formation of casein is attributable to polar amino acid groups (≈ 55%) responsible for the hydrogen bond formations but this property leads to poor electrospinnability arising from low viscoelasticity in solution (113). Addition of suitable synthetic polymers such as polyethylene oxide (113) and polyvinyl alcohol (114) can solve the electrospinning problem. There is a single study on the casein-based topical antimicrobial preparation performed by Selvaraj et al. based on electrospinning of casein nanofibers with silver nanoparticles (113). In this study, the blended mixture of casein, polyethylene oxide, and silver nanoparticles was electrospun to prepare nanofibrous mats that were crosslinked with glutaraldehyde vapor. The incorporation of silver nanoparticles led to the potent antimicrobial activity against *S. aureus, E. coli*, and *B. subtilis*. Further casein-based topical antimicrobial formulations need to be developed to assess their potential in the treatment of topical infections, especially in chronic wounds.

## Protein-Based Delivery Systems for Antibacterial Therapy

Based on the available literature, as presented above, different delivery systems (hydrogels, films, wafers, and electrospun fibers and mats) have been prepared for the antibacterial therapy. An overview of these delivery systems is presented below:

### Hydrogels

Hydrogels are three-dimensional polymeric networks capable of absorbing large amounts of water or biological fluids (up to 1,000 times their dry weight). Their insolubility is attributed to the presence of cross-links between the constituents that form the polymeric network (115, 116). Hydrogels have several advantages including biocompatibility, tunable biodegradability, mechanical strength, and porous structure suitable for topical applications (117). Proteins can be suitable building blocks for hydrogels since they are biocompatible and easily degraded by the body (118). More importantly, different chemical reactions including click chemistry, UV-initiated cross-linking, Michael addition of cysteine residues to vinyl sulfones or maleimides, and native chemical reactions can be performed with proteins to form hydrogels (119). Collagen is widely used as a biomaterial in its native fibrillar form as well as after denaturation to form hydrogel (120). The factors influencing self-assembly of collagen to hydrogel include temperature, ionic conditions, pH, and cross-linking agent (121). Silk fibroin forms β-sheets due to the presence of the highly repetitive amino acid motif GAGAGS. These sheets are responsible for silk fibroin's insolubility in water. The hydrophobic interactions among the protein chains in fibroin result in the assembly of the material into hydrogels. Gelation can be enhanced by an increase in temperature or fibroin concentration, a decrease in pH or by addition of a hydrophilic polymer. Furthermore, addition of Ca^2+^ ions also accelerates the formation of hydrophobic interactions with the COO^−^ ions of the amino acid side chains (122). Different antimicrobials can be incorporated into these protein hydrogels for inducing the antibacterial effects on infected wounds.

### Films

Film dressings are adhesive, durable, comfortable, transparent, and cost-effective materials used for covering the wounds and providing antibacterial effects. Films can be made up of different materials including proteins. Protein films are one of the most widely synthesized protein-based materials. Different strategies are employed in generating protein films which include: natural self-assembly of proteins (123), cross-linking of proteins via physical or chemical approaches (124), and thermal treatment for initiation of the reorganization of proteins (125, 126). Furthermore, post-functionalization of the surface or incorporation of additives can impart new characteristics or enhance native properties of the film (127). Structural proteins with highly repetitive amino acid sequences such as silk and collagen naturally self-assemble into water-stable protein films using different processing methods like casting or printing (123, 128). Silk fibroin films possess properties including biocompatibility, slow degradation, and robust mechanical properties (129, 130). Silk fibroin self-assembles into β-sheets resulting in water-stable films. Mechanical strength and biodegradability of silk fibroin films can be enhanced by controlling β-sheet percentage using different strategies such as methanol annealing, water annealing, and drying speed (131, 132). Collagen consists of three parallel polypeptide-α chains in a right-handed triple helical structure that self-associates to form highly ordered cross-linked fibrils (133) resulting in collagen film insolubility in water. Collagen is usually purified and can be dissolved in acid solutions to form films (134) or alternatively, collagen fibers are directly used to prepare films (135).

### Wafers

Wafers are prepared as freeze-dried or electrospun forms of proteins consisting of porous structures (136). Collagen can be used to form wafers in freeze-dried form, which can absorb fluid upon application to the wound surface and change their state from a dehydrated porous solid to a viscous gel permitting the diffusion of antimicrobial agent to the wounds (137, 138). Silk fibroin porous wafers can be prepared using porogens, freeze-drying, gas forming, and electrospun fibers (139) for application to the wounds.

### Electrospun Fibers and Mats

Electrospinning is a process of applying a high voltage to a polymer solution for transforming a drop at the needle tip into a cone shape in order to generate a jet (140). The ejected jet undergoes a number of instabilities, during which the solvent from the solution is evaporated and dry fibers are collected on the grounded or oppositely charged plates (141). The diameter size, distribution, and morphology of electrospun fibers can be adjusted and tuned according to the solution (e.g., molecular weight, concentration, viscosity, surface tension, conductivity, and dielectric constant) and process parameters (e.g., temperature, flow rate, humidity, working distance, and voltage) (142). Proteins are widely used for the preparation of electrospun wound dressings alone or in combination with other natural and/or synthetic polymers. Zein is extensively utilized in the preparation of electrospun fibers and mats for antimicrobial delivery to infected wounds. A major advantage of electrospinning zein is the avoidance of toxic solvents and cross-linkers due to its solubility in aqueous ethanol and self-assembling nature (143, 144). However, aqueous ethanol is not an ideal solvent for electrospinning (145) because of its high evaporation rate that leads to needle clogging, formation of ribbon-shaped fibers and results in poor water solubility of the fibers (146, 147). This problem can be overcome by co-axial electrospinning with ethanol (146) or polyethylene oxide (PEO) as a shell solution (148) or by cross-linking zein with UV radiation (149). Casein has recently gained attention as a starting material for electrospinning. It's 55% is constituted of polar amino groups which allow the formation of hydrogen bonds. This property leads to poor electrospinnability of casein which is further compromised due to its low viscoelasticity in solution (113, 150, 151). Therefore, electrospinning is only possible upon addition of synthetic polymers such as polyethylene oxide (PEO) (113) and polyvinyl alcohol (PVA) (114). Furthermore, the high hydrophilicity of casein leads to weak mechanical strength and water stability, which requires the use of toxic cross-linking agents such as silane and glutaraldehyde (113, 114). Silk fibroin protein has a low weight (1.3 g cm^−3^) and high tensile strength (~ 4.8 GPa) which makes it ideal for the production of electrospun fibers (152). The mechanical properties of silk fibroin fibers can be altered through methanol treatment after electrospinning, which increases β-sheet crystallinity and reduces water solubility (153). In case of collagen protein, care needs to be taken during electrospinning because denaturation may occur at high voltage (154). Collagen is often co-electrospun with other synthetic polymers such as polycaprolactone (PCL) to increase fiber stability (155).

## Opportunities and Challenges of Protein-Based Systems for Topical Antibacterial Therapy

Proteins possess a number of benefits for topical application in the treatment of infected wounds. The biocompatibility of proteins makes them suitable as a topical formulation for antibacterial therapy (156). Furthermore, the biodegradability of proteins, irrespective of their origin (plant or animal), is possible via physiological mechanisms (157). For example, collagen is naturally degraded in chronic wounds by matrix metalloproteinases (158) and zein is degraded by enzymatic, microbial, or cell phagocytosis pathways (159). Proteins such as collagen, gelatin, and elastin possess cell-recognition motifs like RGD (Arginine-Glycine-Aspartate) that can facilitate recognition by cells to promote cell adhesion and cell migration across the wound bed for promotion of wound healing (160) in addition to their antimicrobial potential. Other proteins such as lactoferrin (161) and lysozyme (38) possess innate antimicrobial, anti-inflammatory, and anti-oxidant activity to facilitate antibacterial and wound healing effects. A great diversity of antimicrobial agents (antibiotics, antimicrobial nanoparticles, antimicrobial peptides, antimicrobial polymers), as mentioned above, can be integrated into protein-based systems to elicit antimicrobial responses against Gram-positive and Gram-negative bacteria.

Besides several benefits, there are still some challenges in the use of protein-based systems for topical antibacterial therapy. The treatment of biofilms on infected wounds is still challenging. Protein based systems can control or sustain the release of antibacterial agent, however, penetration into the biofilm is still limited (162). Therefore, suitable modifications in the protein-based systems (e.g., inclusion of biofilm disassembly agents like nuclease and extracellular protease within the protein formulation) could enhance the antibacterial activity of the system (163). The lack of regulations to ensure structural homogeneity and purity of protein-based materials has led to variations in their quality (164). The purity, composition, and activity of proteins are affected by the diversity in extraction and purification methods that can influence the formulation characteristics. The preparation methods for topical formulations require the use of different solvents (water or organic solvents) with proteins that can affect protein stability and activity (165). Sterilization of the proteins is another challenge (166). Conventional sterilization techniques based on high temperature or gamma radiation have limitations, such as protein denaturation and changes in the amino acid structure (166–168). Proteins can be susceptible to UV radiation as well (169). Viable alternative sterilization methods that can preserve protein structure and properties include supercritical CO_2_, acid treatment (with peracetic acid), and the use of gas plasma (166, 170–172). Most common protein-based systems for topical antibacterial therapy include films, hydrogels, and electrospun matrices. The preparation of films and hydrogels requires a simple blend of proteins with antimicrobial agents and thus does not have a greater impact on the stability and activity of proteins (81, 173). However, the electrospinning of proteins requires the use of different components including cross-linking agents, organic solvents, and a high voltage that can potentially damage protein structure and subsequent loss of its activity (174). Electrospinning of hydrophilic proteins like silk fibroin and casein is also challenging due to its surface tension in water leading to non-continuous processes and artifacts in fibers in addition to their aggregation and low degree of protein unfolding in water (175). Furthermore, for proteins with amphiphilic nature such as zein, the final product loses its fibrous structure and becomes more elastic upon contact with water (148). Therefore, choice of protein and suitable modifications in the formulation methods are necessary to maintain protein's functions and stability as a potent protein-based antibacterial system. So far, there are few reports on the use of different protein-based systems for topical antibacterial therapy. Wide varieties of proteins (plant- and animal-based) besides mentioned in this review are available. However, studies on their use for topical antibacterial therapy are still lacking. Therefore, extensive research on such proteins is warranted to explore their potential as topical antibacterial formulations.

## Conclusions and Future Prospects

Protein-based systems are emerging as potential formulations for topical delivery of antimicrobial agents. The beneficial properties of proteins including biocompatibility, biodegradability, and low immunogenic response make them suitable carriers for different antimicrobial agents. The preparation method of topical formulations (films, hydrogels, and mats) requires different treatment conditions that can affect protein stability and activity. Hence, proper optimization of the preparation method to ensure stability and activity of proteins along with potent antimicrobial delivery is required. Protein-based systems are capable of controlling the release of active ingredients once applied topically to the infected wound and can act as wound protein mimics to support the wound healing process. Furthermore, the ability of proteins to absorb wound exudates and prevent secondary infections makes them suitable candidates for topical application as an antibacterial system for infected wounds. However, treatment of the biofilms is still challenging attributable to limited penetration of the antibacterial agents. Therefore, suitable modifications in the protein-based formulations for enhancing the antibacterial efficacy is warranted. There are several *in vitro* studies performed for the evaluation of the antibacterial activity of protein-based topical antibacterial formulation, however, *in vivo* studies are still lacking. More *in vivo* studies of such potent formulations are required in the future to support the clinical translation of the prepared protein-based systems as topical anti-bacterials.

## Author Contributions

All authors listed have made a substantial, direct and intellectual contribution to the work, and approved it for publication.

## Conflict of Interest

The authors declare that the research was conducted in the absence of any commercial or financial relationships that could be construed as a potential conflict of interest.
